# ASP-Det: Toward Appearance-Similar Light-Trap Agricultural Pest Detection and Recognition

**DOI:** 10.3389/fpls.2022.864045

**Published:** 2022-07-06

**Authors:** Fenmei Wang, Liu Liu, Shifeng Dong, Suqin Wu, Ziliang Huang, Haiying Hu, Jianming Du

**Affiliations:** ^1^Institute of Intelligent Machines, Hefei Institutes of Physical Science, Chinese Academy of Science, Hefei, China; ^2^University of Science and Technology of China, Hefei, China; ^3^Computer Teaching and Research Office of the Department of Information Engineering PLA Army Academy of Artillery and Air Defense, Hefei, China; ^4^Shanghai JiaoTong University, Shanghai, China

**Keywords:** appearance-similarity pest detection, pairwise self-attention, skip-calibrated convolution, object relative size, anchor-free

## Abstract

Automatic pest detection and recognition using computer vision techniques are a hot topic in modern intelligent agriculture but suffer from a serious challenge: difficulty distinguishing the targets of similar pests in 2D images. The appearance-similarity problem could be summarized into two aspects: texture similarity and scale similarity. In this paper, we re-consider the pest similarity problem and state a new task for the specific agricultural pest detection, namely **A**ppearance **S**imilarity **P**est **D**etection (ASPD) task. Specifically, we propose two novel metrics to define the texture-similarity and scale-similarity problems quantitatively, namely Multi-Texton Histogram (MTH) and Object Relative Size (ORS). Following the new definition of ASPD, we build a task-specific dataset named PestNet-AS that is collected and re-annotated from PestNet dataset and also present a corresponding method ASP-Det. In detail, our ASP-Det is designed to solve the texture-similarity by proposing a Pairwise Self-Attention (PSA) mechanism and Non-Local Modules to construct a domain adaptive balanced feature module that could provide high-quality feature descriptors for accurate pest classification. We also present a Skip-Calibrated Convolution (SCC) module that can balance the scale variation among the pest objects and re-calibrate the feature maps into the sizing equivalent of pests. Finally, ASP-Det integrates the PSA-Non Local and SCC modules into a one-stage anchor-free detection framework with a center-ness localization mechanism. Experiments on PestNet-AS show that our ASP-Det could serve as a strong baseline for the ASPD task.

## 1. Introduction

Diversity pest control and prevention are always a crucial agricultural issue worldwide (Sivakoff et al., 2012). To build a cost-effective and efficient pest controlling system, most of the current methods deal with pest monitoring as a pest detection task (Shen et al., 2018). Specifically, the applications employing computer vision techniques attempt to exploit vision features extracted from pre-defined Convolutional Neural Network (CNN) and analyze the visual information to recognize or detect a targeted pest (Deng et al., 2018) and plant leaf disease (Dhaka et al., 2021). Generally, these applications are deployed into a mobile camera or other flexible vision sensors (Liu et al., 2017).

However, in the practical agricultural environment, the in-field pest detection systems require high-quality image resolution and strict image collection standards, e.g., the distance between the camera and pest targets cannot be larger than 1 m (Wang et al., 2021). Besides, these approaches might confront troubles in recognizing lots of pest categories at the same time (Ayan et al., 2020). These limit the functional performance when employing these computer vision algorithms in real-world pest monitoring (Wang et al., 2020). Under this case, several works attempted to install fixed stationary cameras in light traps to monitor pest occurrence by recognizing and detecting the trapped pests (Liu et al., 2019a). But there are two challenges when identifying these captured pests: (1) a large number of pest categories usually share similar textures in images that prevent fine-grained classification. (2) the size of one pest is very close to each other, making it difficult to distinguish them. These challenges are considered appearance-similarity problems in computer vision and pest detection tasks.

In this paper, we pay attention to dealing with the challenges of pest recognition and detection in light traps, which use frequency-vibrating insecticidal lamps to capture pests and use a fixed camera to take pictures of pests that fall into the trapping tray, and stating a new task for the specific agricultural pest detection problem, namely **A**ppearance **S**imilarity **P**est **D**etection (ASPD) task. This task clearly defines and summarizes the appearance-similarity problems from two aspects: texture-similarity and scale-similarity. To further describe these two problems, we define the corresponding metrics: (1) Multi-Texton Histogram(MTH), a statistical index representing the distribution of pests' textures. (2) Object Relative Size (ORS), measuring the pest sizes in captured RGB images. From MTH and ORS, we formulate the ASPD to be a novel pest detection task.

To validate the difficulty of the ASPD task, we build a task-specific dataset, namely PestNet-AS. This dataset is collected and re-annotated from the famous pest detection benchmark PestNet (Liu et al., 2019b). In PestNet-AS, we present a hierarchical category taxonomy. The sup-classes in PestNet-AS are Lepidoptera and Coleoptera, the former contains 17 sub-class categories and the latter contains 7. In total, the PestNet-AS dataset covers 87,672 images and 554,761 pest annotations. Our dataset is aligned with the ASPD task.

Accompanying with ASPD task and PestNet-AS dataset, we propose a deep learning framework ASP-Det to evaluate the performance of the ASPD task. Specifically, our ASP-Det is designed to solve the texture-similarity by submitting a Pairwise Self-Attention (PSA) mechanism and Non-Local Modules to construct a domain adaptive balanced feature module that could provide high-quality feature descriptors. On the other hand, we also present a Skip-Calibrated Convolution (SCC) module that can balance the scale variation among the pest objects and re-calibrate the feature maps into the sizing equivalent of pests. Finally, we constructed a one-stage feature detector for the ASPD task, using a deep convolutional layer of free-anchor. We also introduce a center-ness calibration center strategy for the construction to compensate for the potential localization inaccuracy caused by the absence of the RPN. Finally, this model considers meeting the practical application requirements in agricultural fields.

Our contributions could be summarized as follows:

- We re-consider the light-trap pest recognition and detection problem and state a new pest detection task ASPD. In this task, we quantitatively define the texture-similarity and scale-similarity problems in pest detection using MTH and ORZ metrics.
- We build a new large-scale dataset PestNet-AS specific to ASPD tasks. The dataset contains 87,672 images and 556,521 pest annotations.
- We propose a novel ASP-Det network to address the challenges of the ASPD task. We present PSA mechanism and Non-Local Modules module for dealing with the texture-similarity problem and the SCC module for Scale-Similarity. We believe our ASP-Det could serve as a strong baseline for ASPD tasks and further promote agricultural pest monitoring applications.

## 2. Related Work

### 2.1. Anchor-Free Object Detection

Convolutional neural network-based Object detectors can be divided into two types, namely anchor-based and anchor-free, based on whether anchors are preset. The former can be divided into one-stage and two-stage detection models, and the latter can be divided into key-point-based and center-based detection models. Anchor-free based on keypoint detection algorithms include CornerNet (Law and Deng, 2020), Grid R-CNN (Lu et al., 2020), ExtremeNet (Zhou et al., 2019), and CenterNet (Duan et al., 2019). Anchor-free based on the center point algorithm is a type of detection method that defines the target center point or central area as a positive sample and then regresses the distance from the four sides of the bounding box. YOLO series (Redmon et al., 2016; Bochkovskiy et al., 2020), DenseBox, RetinaNet (Lin et al., 2017b), FCOS (Tian et al., 2019), and FoveaBox (Kong et al., 2020) all belong to this category. Generally, these methods occupy less computing resources and are faster than anchor-based methods. They are suitable for high-speed real-time object detection tasks in applications.

### 2.2. Pest Detection

At present, scholars have studied more general object detection methods. However, these methods cannot be directly utilized in the pest detection tasks, which we confront are relatively particular. Different from pest recognition methods, pest detection methods based on the deep learning methods used deep convolutional networks (Dai et al., 2016) to automatically identify the category and location of the target according to the model algorithm. Liu et al. (2019b) put forward an approach for large-scale multi-class pest detection, which can detect 16 classes of agricultural pests using an End-to-End deep convolutional neural network. Jiao et al. (2020) proposed a two-stage anchor-free convolutional neural network to realize small-scale pests detection for the multi-categories agricultural pest. Yao and Xu (2020) proposed an automatic detection model for pest damage symptoms on rice canopy based on improved RetinaNet. The average accuracy of the detection of the two pests in the pest-like area reached 93.76%. Dan et al. (2021) showed a method of automatic greenhouse insect pest detection and recognition based on a cascaded deep learning classification. Tetila, EC. used five deep learning architectures with a fine-tuning for the category of soybean pest images, which reached an accuracy of up to 93.8% (Tetila et al., 2020). Wang. et al. integrated context-aware information representation in-field. A multi-projection pest detection model (MDM) was proposed and trained by crop-related pest images in Wang et al. (2020). Automatic in-trap pest detection by end-to-end on a GPU workstation with data augmentation and then deployed on embedded devices with minimal prepossessing in Sun et al. (2018).

### 2.3. Similar Object Detection

Similar object detection considers detection methods with more detailed features. The general approaches adopt fine-grained strategies to address the challenges. The current research on fine-grained detection mainly includes the following content: Feng (2013) proposed a set of training images, which can identify a sparse number of image patches in the training set which cover most parts of the target object in the test image. Li et al. (2016) used fine-grained detection for face-screen distance on smartphones. However, there are only a few applications of fine-grained detection related to agriculture and almost few for similar pest detection. Thus, this paper conducts a detailed study on the feature extraction of similar pests, builds a model, and provides an algorithm framework with better accuracy and real-time performance.

## 3. Problem Statement

We present the Appearance-Similarity Pest Detection(ASPD) task in our work. Specifically, we define ASPD task from two aspects: texture-similarity that describes the gray-level and color-level appearance of these pest targets (Section 3.1), and scale-similarity that describes size-level appearance of pests (Section 3.2). For each problem, we propose the corresponding metrics to define these settings.

### 3.1. Texture-Similarity

To quantitatively define texture-similarity, we consider it from the following: (1) gray-level similarity that defines whether the objects are similar in gray images. (2) color-level similarity that defines whether the colorized pests are similar.

For gray-level similarity, a Hash algorithm is a common method to describe image similarity. In detail, the perceptual Hash (pHash) algorithm usually achieves better performance than deference Hash (dHash) as well as average Hash (aHash). Thus, we propose to use the pHash to analyze and define the gray-level similarity problem. In this metric, we randomly select 100 images from one category of pest, calculate 32 × 32 Discrete Cosine Transform (DCT), and select 8 × 8 matrix in the upper left corner. Next, we apply pHash algorithm to extract the pest target representation value, as the object gray-level representation. Finally, we define the object similarity such that the representation value is larger than 0.6.

On the other hand, we consider color-level pest similarity. In this problem, we first use MTH to describe the repetition law and repetition mode of the image pixel-level information, expressed in texture information in different color spaces. In terms of texture information, the multi-element histogram method uses the Sobel operator to detect the edge of the image and detect the texture direction and then describes the texture and shape information of the image. The Sobel operator calculates the three color channels separately in the RGB color space. The two vectors corresponding to the horizontal and vertical directions are returned in each channel. *a*(*R*_*x*_, *G*_*x*_, *B*_*x*_) and *b*(*R*_*y*_, *G*_*y*_, *B*_*y*_) represent the gradient information in the corresponding direction of the corresponding channel. Further, we can obtain the texture by calculating formulas 1–4.


(1) $$\begin{array}{l} |a|=\sqrt{{\left(R_{x}\right)}^{2}+{\left(G_{x}\right)}^{2}+{\left(B_{x}\right)}^{2}} \end{array}$$

(2) $$\begin{array}{l} |b|=\sqrt{{\left(R_{y}\right)}^{2}+{\left(G_{y}\right)}^{2}+{\left(B_{y}\right)}^{2}} \end{array}$$

(3) $$\begin{array}{l} a\cdot b=R_{x}\cdot R_{y}+G_{x}\cdot G_{y}+B_{x}\cdot B_{y} \end{array}$$

(4) $$\begin{array}{l} \theta =\text{arccos}\left[\frac{a\cdot b}{|a|\cdot |b|}\right] \end{array}$$

In terms of color information, the results obtained from the three channels of R, G, and B are quantified into 64 color images with four different primitives in *C*(*x, y*). Perform texture detection in the process to obtain the texture primitive image *T*(*x, y*). Finally, according to *T*(*x, y*), a multi-element histogram describes texture features. The definition of the MTH is shown in formulas 5 and 6:


(5) $$\begin{array}{l} H\left(T\left(P_{1}\right)\right)=N\left\{\theta \left(P_{1}\right)=v_{1}⋀\theta \left(P_{2}\right)=v_{2}\|P_{1}-P_{2}\|=D\right\} \end{array}$$

(6) $$\begin{array}{l} H\left(T\left(P_{1}\right)\right)=\bar{N}\left\{\theta \left(P_{1}\right)=w_{1}⋀\theta \left(P_{2}\right)=w_{2}\|P_{1}-P_{2}\|=D\right\} \end{array}$$

where *P*1 = (*x*_1_, *y*_1_), *P*2 = (*x*_2_, *y*_2_) represent two adjacent pixels with a distance of D in the original image. Their corresponding pixels in the primitive image *T*(*x, y*) are *T*(*P*1) = *w*_1_ and *T*(*P*2) = *w*_2_, respectively. In the texture direction matrix θ(*x, y*), the directions of the points P1 and P2 are θ(*P*1) = *v*_1_, θ(*P*_2_) = *v*_2_. N represents the number of times *v*_1_ and *v*_2_ appear together, and $\bar{N}$ represents the number of times *w*_1_ and *w*_2_ appear together. *H*[*T*(*P*1)] represents the number of times that the same edge direction appears at the same time under a certain color background; it represents the number of times the same color appears under a certain edge direction. Therefore, the texture feature vector *f*_*v*_ of the image is expressed as shown in formula 7:


(7) $$\begin{array}{l} \text{f}\left(v\right)=H\left(T\left(P_{1}\right)\right)◦H\left(\theta \left(P_{1}\right)\right) \end{array}$$

where ° means connection.

The similarity of images *I*_1_ and *I*_2_ is defined as shown in Equation (8):


(8) $$\begin{array}{l} S_{I}\left(I_{1},I_{2}\right)={\left\|f_{v}\left(I_{1}\right)-f_{v}\left(I_{2}\right)\right\|}^{-1} \end{array}$$

where ‖*f*_*v*_‖ denotes Euclidean distance.

### 3.2. Scale-Similarity

We adopt ORS to measure the problem for scale-similarity. Specifically, given an RGB image with a shape of *H* × *W* and the *i*-th pest bounding box *H*_*i*_ × *W*_*i*_, the ORS_*i*_ of this pest object is defined as follows:


(9) $$\begin{array}{l} {\text{ORS}}_{i}=\frac{H_{i}\cdot W_{i}}{H\cdot W} \end{array}$$

In this way, we can count the ORS for the *c*-th category in the entire dataset by


(10) $$\begin{array}{l} {\text{ORS}}^{\left(c\right)}=\frac{\sum_{i=1}^{M}ORS_{i}\cdot \text{sgn}\left(c_{i},c\right)}{\sum_{i=1}^{M}\text{sgn}\left(c_{i},c\right)} \end{array}$$

where *M* is the number of pest objects and function sgn(·) indicates whether the category of *i*-th pest is *c*-th class, that belongs to defined as


(11) $$\begin{array}{l} \text{sgn}\left(c_{i},c\right)=\{\begin{array}{cc} 1 & \;c_{i}=c\\ 0 & \;c_{i}\neq c \end{array} \end{array}$$

**Figure 1:**
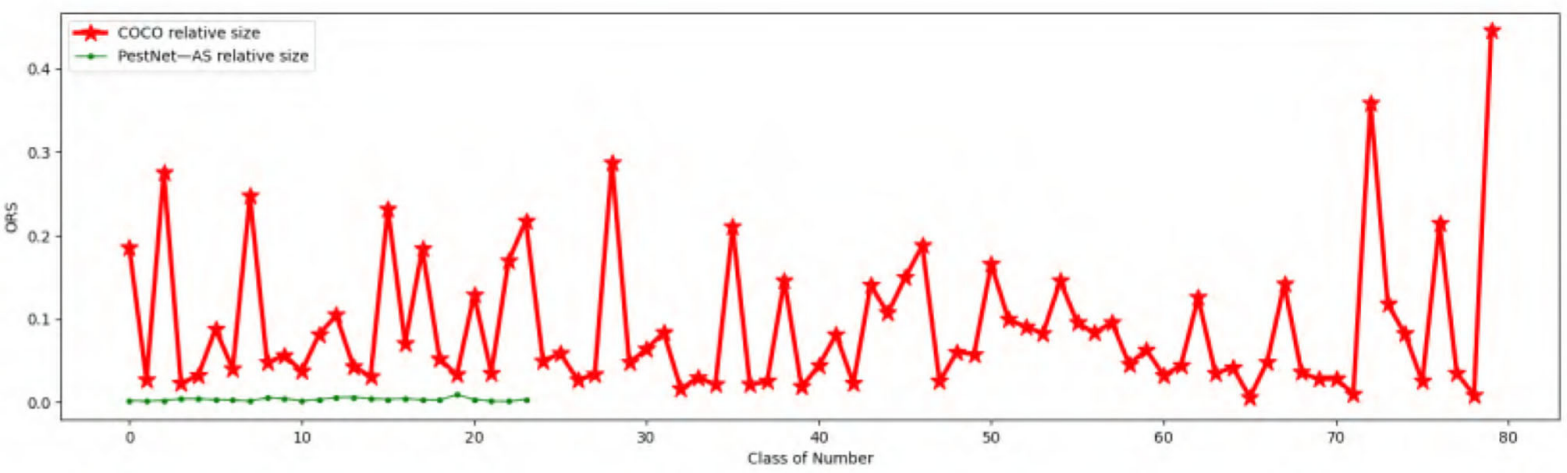
Comparison of Object Relative Size (ORS) with common object datasets MS COCO and PestNet-AS.

Finally, we can obtain the ORS distribution map of all the categories of pest species. Figure 1 illustrates the Relative Size distribution of our targeted 24 pest categories. All the ORS of all pest objects are not larger than 1%, which indicates that all the pests in our work are small in size. Furthermore, most of the categories hold nearly 0.5% ORS, which is in line with the difficulty of scale-similarity in the ASPD task.

## 4. Dataset

To solve the ASPD task, we present a large-scale dataset named PestNet-AS, which is built from a popular dataset PestNet (Section 4.1). To meet the ASPD problem setting, we analyze our PestNet-AS dataset from texture-similarity and scale-similarity (Section 4.2).

### 4.1. Data Collection

**Figure 2:**
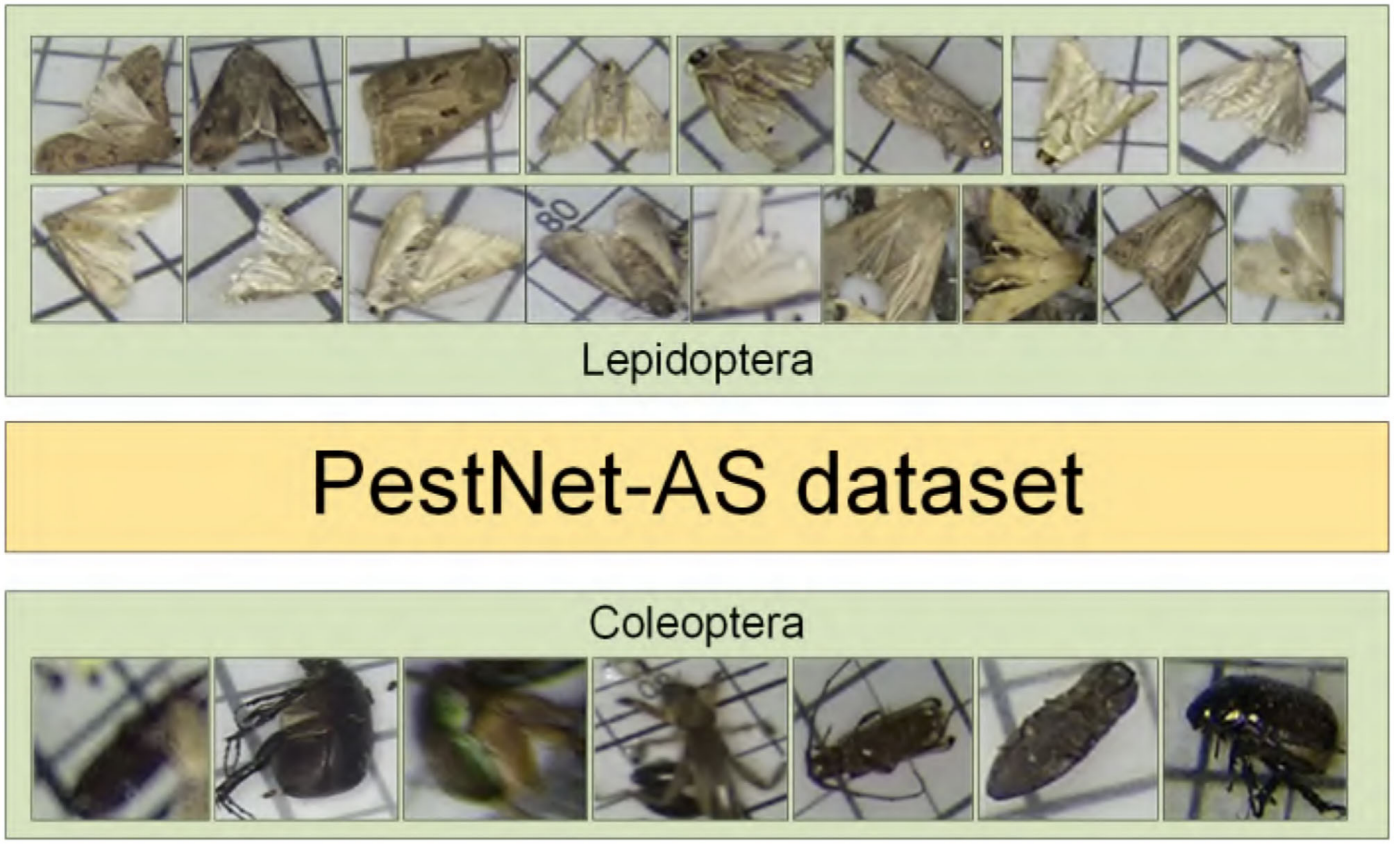
Visualization of two sup-class of pests: the figure shows the visualization of similar pests in the 17 sub-classes of the Lepidoptera and 7 sub-classes of Coleoptera.

To the best of our knowledge, there is no dataset suitable for the similarity pest detection task, so we extract a sub-dataset with a similar appearance from PestNet, filter, and re-annotate it. We select part of the categories of PestNet to validate our PestNet-AS task and method. Specifically, we build a simple category taxonomy, as shown in Figure 2. The taxonomy contains 2 sup-classes and 24 sub-classes(categories).

This paper resizes these pest images to 1,333 × 800 from 2,560 × 1,920 and 2,592 × 1,944. We chose 87,672 pictures and divided into two sup-classes and 24 sub-classes. Table 1 shows two categories of pests' scientific names, their average relative size to the whole pest images. The two significant pest portraits are shown in Figure 1.

**Table 1:** Description of pests of the two sup-classes.

**Pest ID**	**Sup-class**	**Sub-class**	**No. of images**	**No. of instances**	**ORS (%)**
1	Lepidoptera	Spodoptera frugiperda	226	241	0.189
2		Rice leaf roller	7,430	12,994	0.124
3		Chilo suppressalis	3,323	8,462	0.206
4		Xestia c-nigrum	1,691	2,224	0.397
5		Mythimna separata	12,502	25,526	0.403
6		Helicoverpa armigera	25,364	74,769	0.293
7		Ostrinia furnacalis	19,536	43,316	0.238
8		Proxenus lepigone	24,041	122,509	0.144
9		Agrotis exclamationis	1,082	1,782	0.530
10		Spodoptera litura	8,083	10,936	0.448
11		Spodoptera exigua	14,615	28,133	0.151
12		Stem borer	5,719	8,475	0.306
13		Agrotis ipsilon	9,944	15,397	0.567
14		Land cutworms	1,131	1,805	0.601
15		Cabbage moth	7,108	10,410	0.434
16		Scotogramma trifolii Rottemberg	13,114	23,301	0.346
17		Yellow cutworms	3,825	4,933	0.434
18	Coleoptera	Holotrichia parallela	24,041	122,509	0.286
19		Anomala corpulenta	1,082	1,782	0.240
20		Gryllotalpa orientalis	8,083	10,936	0.904
21		Pleonomus canaliculatus	14,615	28,133	0.323
22		Agriotes fuscicollis miwa	5,719	8,475	0.130
23		Melanotus caudex	9944	15,397	0.101
24		Holotrichia oblita	1,131	1,805	0.320

Data annotation was done by professionals using Labeling software under the guidance of entomologists^1^. The pest location coordinates and classes are saved as an XML file, then converted to JSON format, which has the same format as COCO. The number of annotations corresponds to the number of bounding boxes labeled in each image. Every image could contain more than one annotation depending on the number and classes of pests. To evaluate the effectiveness and practicability of the model, we randomly selected images from the dataset according to the proportion of 80% (70,138 images) of the training set and 20% (17,534 images) of the test set.

### 4.2. Dataset Analysis

The PestNet-AS dataset is established to solve the ASPD task, thus it is built to meet the definitions of texture-similarity and scale-similarity problems. We use the designed metric to validate the dataset characteristics on texture-similarity. Concerning gray-level similarity, we apply the pHash algorithm described above to evaluate the 24 sub-classes in the two sup-classes. The results are shown in Tables 2, 3. Almost all pest similarities are more extensive than 0.6, which aligns with the gray-level pest similarity problem definition, which indicates that the pest objects in our PestNet-AS are highly similar in texture.

**Table 2:** Description of the 17 sub-classes of phash 32 × 32 similarity of pests.

	**1**	**2**	**3**	**4**	**5**	**6**	**7**	**8**	**9**	**10**	**11**	**12**	**13**	**14**	**15**	**16**	**17**
1	70.01	–	–	–	–	–	–	–	–	–	–	–	–	–	–	–	–
2	69.74	68.16	–	–	–	–	–	–	–	–	–	–	–	–	–	–	–
3	68.06	68.46	68.53	–	–	–	–	–	–	–	–	–	–	–	–	–	–
4	68.47	65.77	69.26	72.18	–	–	–	–	–	–	–	–	–	–	–	–	–
5	70.84	69.61	71.57	72.36	73.61	–	–	–	–	–	–	–	–	–	–	–	–
6	70.57	69.66	71.47	70.41	71.85	75.34	–	–	–	–	–	–	–	–	–	–	–
7	72.76	71.35	73.17	70.01	72.97	72.97	75.34–	–	–	–	–	–	–	–	–	–	–
8	70.62	68.69	70.45	72.26	71.87	72.94	75.34	77.74	–	–	–	–	–	–	–	–	–
9	69.19	66.68	67.45	70.54	67.98	71.60	72.33	73.96	66.61	–	–	–	–	–	–	–	–
10	70.44	67.85	69.91	69.90	69.69	71.58	74.17	75.16	65.17	70.30	–	–	–	–	–	–	–
11	70.43	67.57	69.61	71.20	71.19	72.26	74.82	77.40	66.53	70.75	65.88	–	–	–	–	–	–
12	71.33	69.33	71.57	69.66	72.34	71.46	76.66	76.11	68.26	69.79	67.92	72.16	–	–	–	–	–
13	69.02	66.89	68.93	71.34	70.07	72.97	72.50	75.81	66.40	70.56	65.83	72.11	72.56	–	–	–	–
14	70.82	68.73	69.35	73.30	70.36	74.34	74.43	76.80	68.26	71.61	67.10	72.16	72.81	72.07 –	–	–	–
15	69.54	67.71	69.50	72.28	70.42	73.32	73.22	76.80	67.35	70.36	67.07	72.68	73.28	71.19	74.12–	–	–
16	71.61	68.92	71.07	72.53	71.96	74.54	76.19	78.33	67.09	71.84	67.80	74.33	73.56	73.19	76.04	74.41	–
17	70.03	68.66	71.03	70.97	71.97	71.25	76.75	76.13	66.21	70.72	67.52	71.68	69.94	70.37	73.86	71.77	68.95

**Table 3:** Description of the 7 sub-classes of phash 32 × 32 similarity of pests.

	**18**	**19**	**20**	**21**	**22**	**23**	**24**
18	62.23	–	–	–	–	–	–
19	62.45	60.85	–	–	–	–	–
20	63.04	62.14	66.07	–	–	–	–
21	63.10	63.17	67.25	68.18		–	–
22	62.45	63.89	66.96	70.05	68.68	–	–
23	62.42	63.64	68.64	68.42	66.92	71.41	–
24	61.75	62.23	66.93	67.21	68.02	72.45	70.63

**Figure 3:**
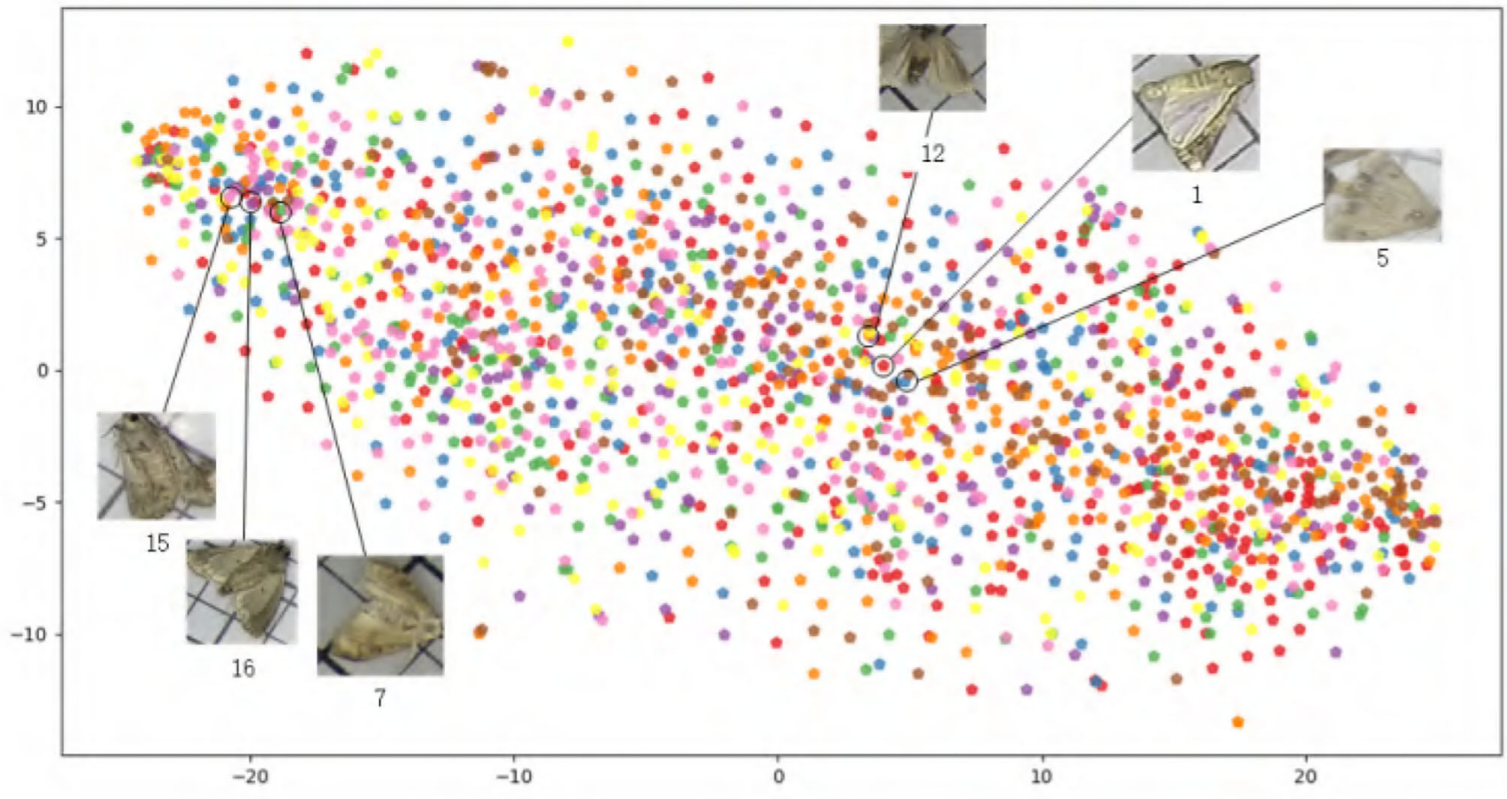
PestNet-AS similarity description in Multi-Texton.

In terms of color-level similarity, we adopt the MTH algorithm to evaluate PestNet-AS dataset. Specifically, we crop all the pest targets in our dataset and calculate their MTH features. Figure 3 shows the t-SNE map on these features. These pests from various categories lie in very close feature spaces and have identical characteristics. Therefore, our PestNet-AS meets the requirement of texture similarity.

For the scale-similarity problem, we calculate ORS for each pest object, and the results are shown in Figure 1. Due to the specific attribute of each object class, the ORS of labeled instances are unevenly distributed among these categories for MS COCO (Lin et al., 2014). Compared with MS COCO, the ORS for our dataset PestNet-AS holds a similar scale for almost all the types, which indicates that our PestNet-AS also meets the scale-similarity problem. Therefore, we can conclude that PestNet-AS could be used as a benchmark for ASPD tasks.

## 5. ASP-Det, A Deep Learning Framework for ASPD

### 5.1. Motivation

**Figure 4:**
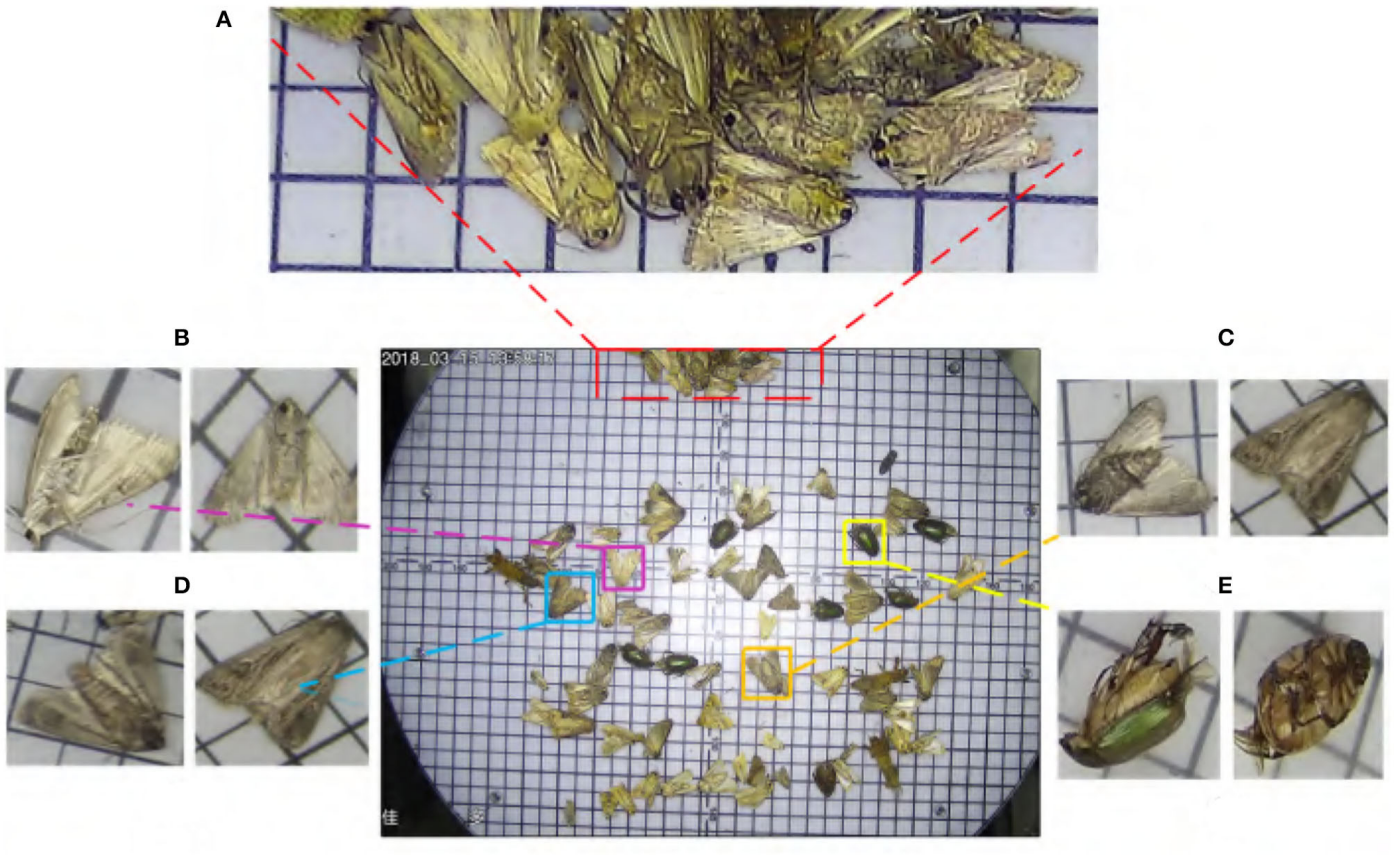
Some typical challenges in appearance-similar pest detection **(A)** appearance-similar pest density distributed; **(B,C)** pests with high similarity on the ventral and dorsal sides; **(D,E)** different postures of appearance-similar pests of the Lepidoptera and Coleoptera.

In this paper, we aim to solve the problem of pests with similar-appearance and size equivalent, which is one of the major challenges in the fine-grained detection task. Specifically, the Pest classification problem is worse than detection. We pay more attention to developing practical pest monitoring systems for appearance-similar pest datasets in light-trap (PestNet-AS). As shown in Figure 4, PestNet-AS contains many challenging issues for pest detection approaches, such as pest targets with dense occlusion, high similarity, including texture similarity and scale similarity. In addition, the relative size of our similar dataset is also smaller than that of the COCO dataset, as shown in Figure 1. Given these thorny problems, we must consider both the detection accuracy and real-time characteristics. Therefore, we propose to use a one-stage pyramid feature extraction model to detect ASPD tasks. The SCC module and the non-local module are added to the model to solve the problem of scale similarity and texture similarity.

#### 5.1.1. Pest Recognition on Texture-Similarity Problem

In the process of pests in the ASPD task, it is not easy to accurately classify because the appearance and texture are too similar. The main reason is that the feature expression is not strong enough. The current method only considers the low-level feature maps in the feature pyramid as their local features. It ignores the high-level semantic information so that the pest targets have sound positioning effects, but classification accuracy is not good. On the other hand, simultaneously considering the simple superposition of low-level and high-level feature map information will cause confusion on local characteristics of pests. Lack of pertinence for pests with high similarity will affect the recognition effect and cause the detection method to be inaccurate. The classification results are shown in Table 4.

**Table 4:** Classification results of appearance-similar pests using different methods.

**Methods**	**Top-1 (%)**	**Top-5 (%)**
ResNet-50	50.2	71.1
SENet	58.6	75.6
VGG-16	48.6	73.7
Inception	42.3	62.8

#### 5.1.2. Pest Detection on Scale-Similarity Problem

The pest scales are too close, and a large number of redundant anchors are not used, which seriously affects the positioning of the frame, so the detection is not very accurate. First, we investigate the network performance in the standard feature pyramid network algorithm. The primary purpose is to express various dimensional characteristics for objects of different sizes effectively. However, the relative scale of our dataset changes little, and the appearance features are incredibly similar. So, the recall rate is not satisfactory at all stages of the IOU. Especially when the IOU becomes more prominent, the recall rate decays more severely. The results are shown in the following Table 5. Considering the characteristics of the PestNet-AS dataset, we expect to use the feature extraction of the feature pyramid network in the model training. To avoid the poor effect caused by small size changes, we need to reconstruct the feature pyramid.

**Table 5:** Recall performance: FCOS on PestNet-AS with ResNet-50-FPN as a backbone.

**IoU**	**0.5**	**0.55**	**0.6**	**0.65**	**0.7**	**0.75**	**0.8**	**0.85**	**0.9**	**0.95**
Recall_1_	0.356	0.337	0.317	0.278	0.254	0.231	0.171	0.112	0.051	0.001
Recall_10_	0.472	0.431	0.415	0.356	0.314	0.251	0.192	0.163	0.082	0.003
Recall_100_	0.614	0.585	0.462	0.382	0.366	0.341	0.275	0.195	0.123	0.007
MRecall	0.588	0.513	0.426	0.365	0.344	0.313	0.254	0.182	0.091	0.005

### 5.2. ASP-Det Overview

**Figure 5:**
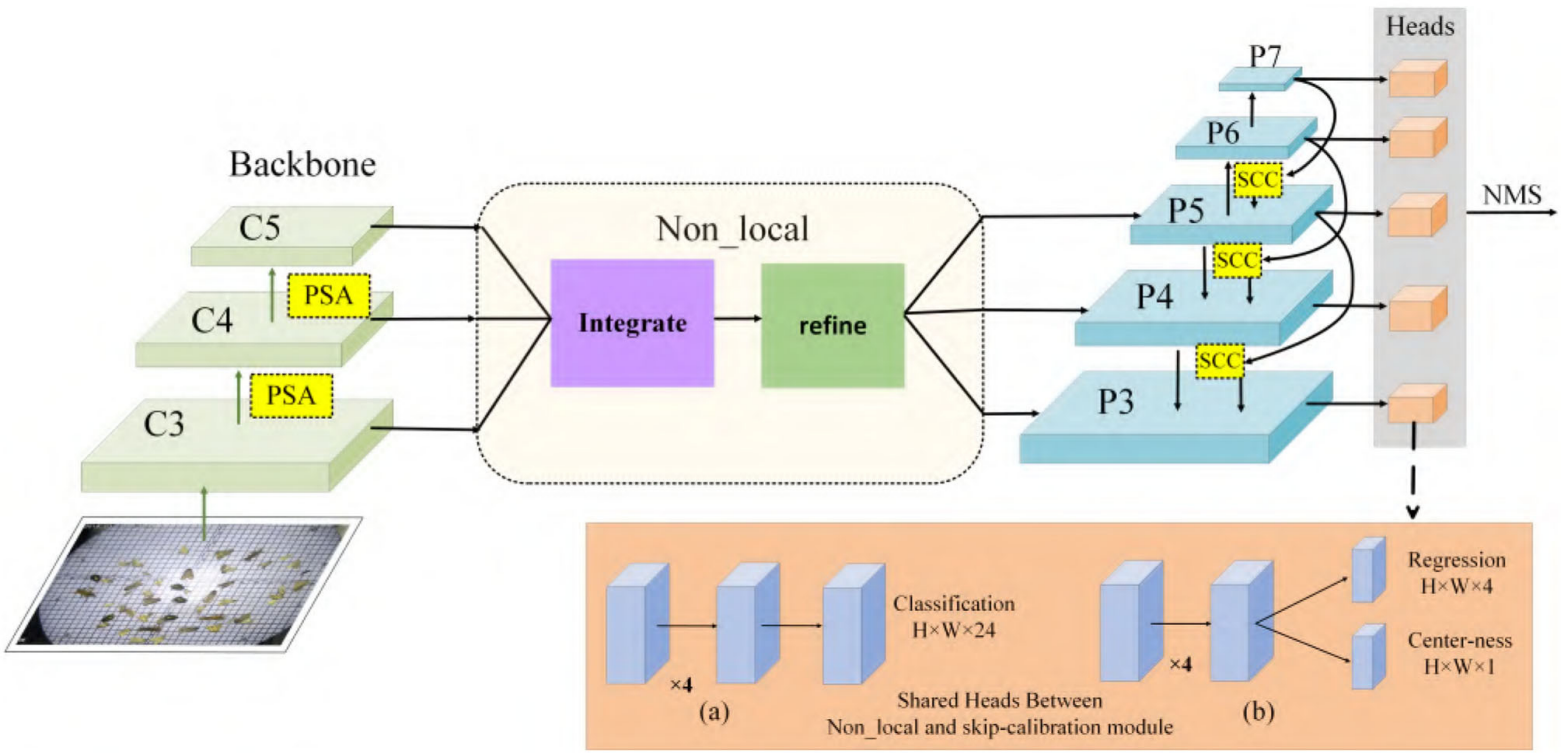
Overview of ASP-Det framework. (a) Classification branch, (b) regression and center branch. PSA, pairwise self-attention module; SCC, skip-calibrated convolution module.

This section describes the proposed scale-calibrated free anchor CNN detection method for appearance-similar agricultural pests. The proposed pest detection model ASP-Det consists of pest features extraction network multi-classes pest detection network. We construct a non-local feature pyramid network (NFP). We construct ASP-Det with PSA module, which can fuse the features with different levels. Then joint skip-calibrated convolution module (SCC) in the features pyramid network for detecting similar pest object. Overview of ASP-Det framework shown in Figure 5. Specifically, we first fed a picture entering the CNN feature extraction network, and we added the PSA channel module during the feature extraction process. Second, a non-local operation is performed on the obtained feature map and then input into the feature pyramid network. Finally, we design an SCC strategy that takes an interval in the feature pyramid to form a feature sampling layer, ensuring the integration of sample features across levels. Third, we introduce center-ness to suppress the low-quality detected bounding boxes produced by the locations far from the center of an object. Finally, non-maximum suppression (NMS) algorithm is employed to remove redundant boxes for the same object (Symeonidis et al., 2019).

### 5.3. PSA Module

Because the dataset has large similarity in appearance and morphology and the number of samples of various classes is not balanced. This paper designs a new feature pyramid that joins the non-local and SCC Modules to resolve the above problems. Different from former approaches (Lin et al., 2017a; Yu et al., 2021) that integrate multi-level features using lateral connections, our key idea is to strengthen the multi-level features using the same deeply integrated balanced semantic features. Each layer simultaneously realizes two functions in CNN, feature aggregation and feature transformation. The former incorporates the characteristics of all positions extracted by the kernels, and the latter performs conversion through linear mapping and nonlinear scalar functions. Thus, the integration function is suitable for phase detection networks, and the transformation function is ideal for feature pyramid networks. Suppose the feature transformation is set as an element-level operation composed of linear mapping and nonlinear scalar functions. In this paper, we introduce the Pairwise module (Zhao et al., 2020) to establish feature aggregation. Consistent with global activated PSA modules, the final result is expressed as a weighted sum of adaptive weights and features:


(12) $$\begin{array}{l} y_{i}=\underset{j\in R\left(i\right)}{\sum }\alpha \left(x_{i},x_{j}\right)⊙\beta \left(x_{j}\right) \end{array}$$

Where *x*_*i*_ and *x*_*j*_ are feature maps with indexes i and j, ⊙ is the Hadamard product called aggregation with the local footprint R(i), several parameters in the PSA module will not be affected by the size of the footprint. After this aggregation, the result *y*_*i*_ can be obtained.

The vectorβ(*x*_*j*_) generated by the function β(·) will be aggregated with the adaptive vector α(*x*_*i*_, *x*_*j*_) introduced later. Compared with ordinary weights, adaptive vector α(*x*_*i*_, *x*_*j*_) has strong content adaptability. It can be decomposed as follows:


(13) $$\begin{array}{l} \alpha \left(x_{i},x_{j}\right)=\gamma \left(\delta \left(x_{i},x_{j}\right)\right) \end{array}$$

where δ(·) and γ(·), respectively, represent a relation function and a hybrid map composed of linear and nonlinear functions. Based on the relation δ(·), the function γ(·) is used to obtain a vector result, which can be combined with β(*x*_*j*_) in Equation (10). In general, matching the output dimension of γ(·) with the dimension of β(*x*_*j*_) is unnecessary because attention weights can be shared among a group of channels. We choose the subtraction as the relation function, which can be formulated:


(14) $$\begin{array}{l} \delta \left(x_{i},x_{j}\right)=\varphi \left(x_{i}\right)-\phi \left(x_{j}\right) \end{array}$$

where φ(·) and ϕ(·) are convolution operations matching output dimensions. δ(·) calculates spatial attention for each channel instead of sharing between channels. We adopt a non-local refine the feature as a pyramid network after aggregation.

Non-local mean (Wang et al., 2017) is a classical filtering algorithm that computes a weighted mean of all pixels in an image. It allows distant pixels to contribute to the filtered response at a location based on patch appearance similarity. The non-local behavior in Equation (15) is because all positions [∀(*j*)] are considered in operation. A convolutional process sums up the weighted input in a local neighborhood as a comparison. A non-local process is a flexible building block that can be used with convolutional layers. It can be added into the earlier part of deep neural networks, unlike *fc* layers that are often used in the end, which allows us to build a hierarchical model that combines non-local and local information.


(15) $$\begin{array}{l} y_{i}=\frac{1}{C\left(x\right)}\underset{\forall \left(j\right)}{\sum }f\left(X_{i},X_{j}\right)g\left(X_{j}\right) \end{array}$$

**Figure 6:**
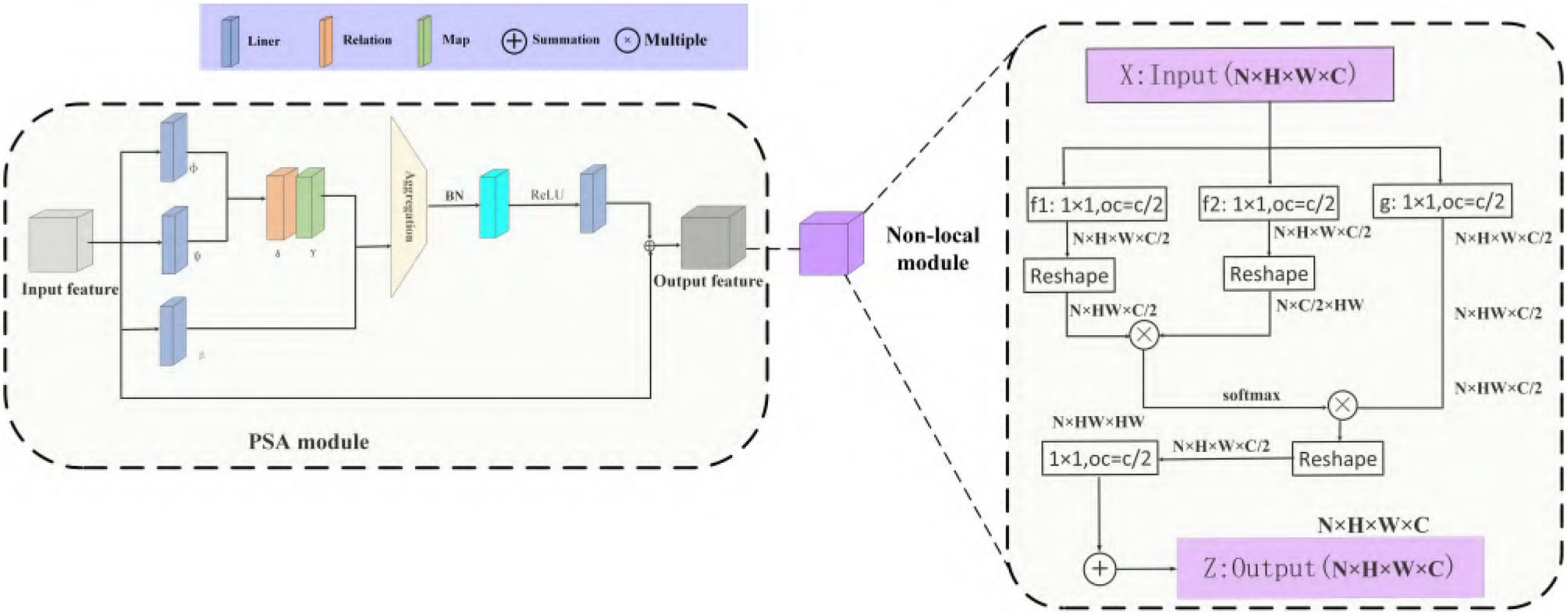
PSA module and non-local module.

The above PSA module uses novel vector attention, which can generate content adaptation ability while maintaining the channel adaptation ability. PSA module makes our appearance-similar target detection model have strong adaptability, which can effectively enhance the salient differences between different features. The pipeline is shown in Figure 6. It consists of two branches and four steps: re-scaling, integrating, refining, and strengthening.

Also, we observe that the similar pests in the images are primarily small and size equivalent. Using state-of-the-art object detection approaches to these images will make similar pest features prone to lose after high-level convolution. It is challenging to extract similar pest features in the network. Hence, the novel Skip-Calibrated Convolution model can combine the delicate features in a high-level convolutional layer. The integral structure of pest come from a low-level convolutional layer. Then, we could fuse the contextual information around pests from the low-level convolutional layer and address the issue of features misjudged for the similar object in the deep convolution layer. In the next section, we will present the alternative optimization for similar pest detection from the internal structure of a CNN and give details of the ASP-Det.

### 5.4. SCC Module

**Figure 7:**
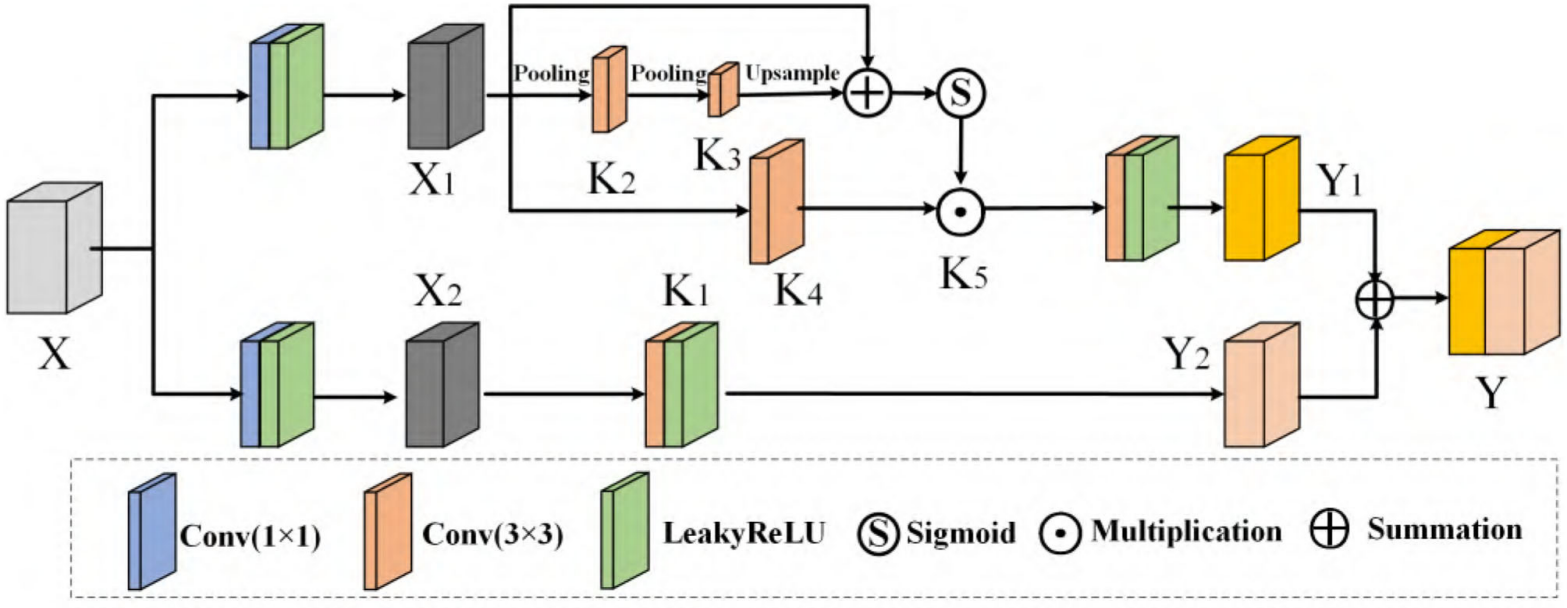
SCC module.

The structure of deep CNNs is becoming more and more complicated, which can enhance the network's learning ability. The novel module called SCC considers improving the feature transformation process in convolution since pests with high similarity may be difficult to judge in adjacent layers. We do not only use the features of the upper layer to perform up-sampling directly but also introduce the information of the following high-level into the sampling so that features have better recognition, adding a specific architecture in Figure 7.

A given group of filter sets K with the shape (C, C, kxh, kxw) is divided into two branches, which are responsible for conducting [K1, K2, K3, K4, K5] different functions, respectively. In SCC, we perform feature transform at two scales: the original scale and the smaller scale after down-sampling. For a given X, we adopt max pooling to reduce the scale:


(16) $$\begin{array}{l} M_{1}=MaxPool_{r}\left(X1\right) \end{array}$$

(17) $$\begin{array}{l} T_{1}=MaxPool_{r}\left(M_{1}\right) \end{array}$$

where *r* is the down-sampling rate and stride of the pooling process. The receptive field at each spatial location can be effectively expanded by benefiting from the down-sampling operation. Next, *T*_1_ can be used as an input to the filter K2 and K3 following the up-sample procedure, which restores the feature to the original scale, resulting in


(18) $$\begin{array}{l} X_{1}^{\prime }=Up\left(F_{2}\left(T_{1}\right)\right)=Up\left(T_{1}\times K_{2}\right) \end{array}$$

(19) $$\begin{array}{l} X_{1}^{\prime \prime }=Up\left(F_{3}\left(X_{1}^{\prime }\right)\right)=Up\left(X_{1}^{\prime }\times K_{3}\right) \end{array}$$

where *F*_2_(*T*_1_) = *T*_1_ × *K*_2_, $F_{3}\left(X_{1}^{\prime }=X_{1}^{\prime }\times K_{3}\right)$ is a simplified form of convolution. Then, the calibrated operation can be formulated as


(20) $$\begin{array}{l} Y_{1}^{\prime }=F_{4}\left(X_{1}\right)⊙Sigmod\left(X_{1}^{\prime \prime }\right) \end{array}$$

Where *F*_4_(*X*_1_) = *X*_1_ × *K*_4_, *Sigmoid*(·) is an activation function. The final result of the skip-calibrated part is calculated:


(21) $$\begin{array}{l} Y_{1}=F_{5}\left(Y_{1}^{\prime }\right) \end{array}$$

Where $F_{5}\left(Y_{1}^{\prime }\right)=Y_{1}^{\prime }\times K_{2}$. The other part can be obtained from another branch that does not require scale transformation. The formula is as follows:


(22) $$\begin{array}{l} Y_{2}=F\left(X_{2}\right)\times K_{1} \end{array}$$

Finally, we sum *Y*_1_ and *Y*_2_ to get the final result Y. Reviewing the entire SCC enables each spatial position to adaptively encode the context from a long-range region, which is also a vast difference between it and the traditional FPN network.

### 5.5. Optimization

ASP-Det is a fully convolutional one-stage object detector. Unlike anchor-based sensors, which consider the location on the input image as the center of anchor boxes and regress the target bounding box for these anchor boxes, we directly revert the target bounding box for each location. Let $F_{i}\in R^{H\times W\times C}$ be the feature maps at layer i of a backbone CNN. For each location(*x, y*) on the feature map *F*_*i*_, we can map it back onto the input image as $\left(\frac{S}{2}+xs,\frac{S}{2}+ys\right)$, which is near the center of the receptive field of the location(*x, y*). Besides the label for classification, we also have a 4D ground truth vector *q* = (*l, r, t, b*) being the regression target for each sample. Here l, r, t, and b are the distances from the location to the four sides of the bounding box. If a location falls into multiple bounding boxes, it is considered an ambiguous sample.

**Figure 8:**
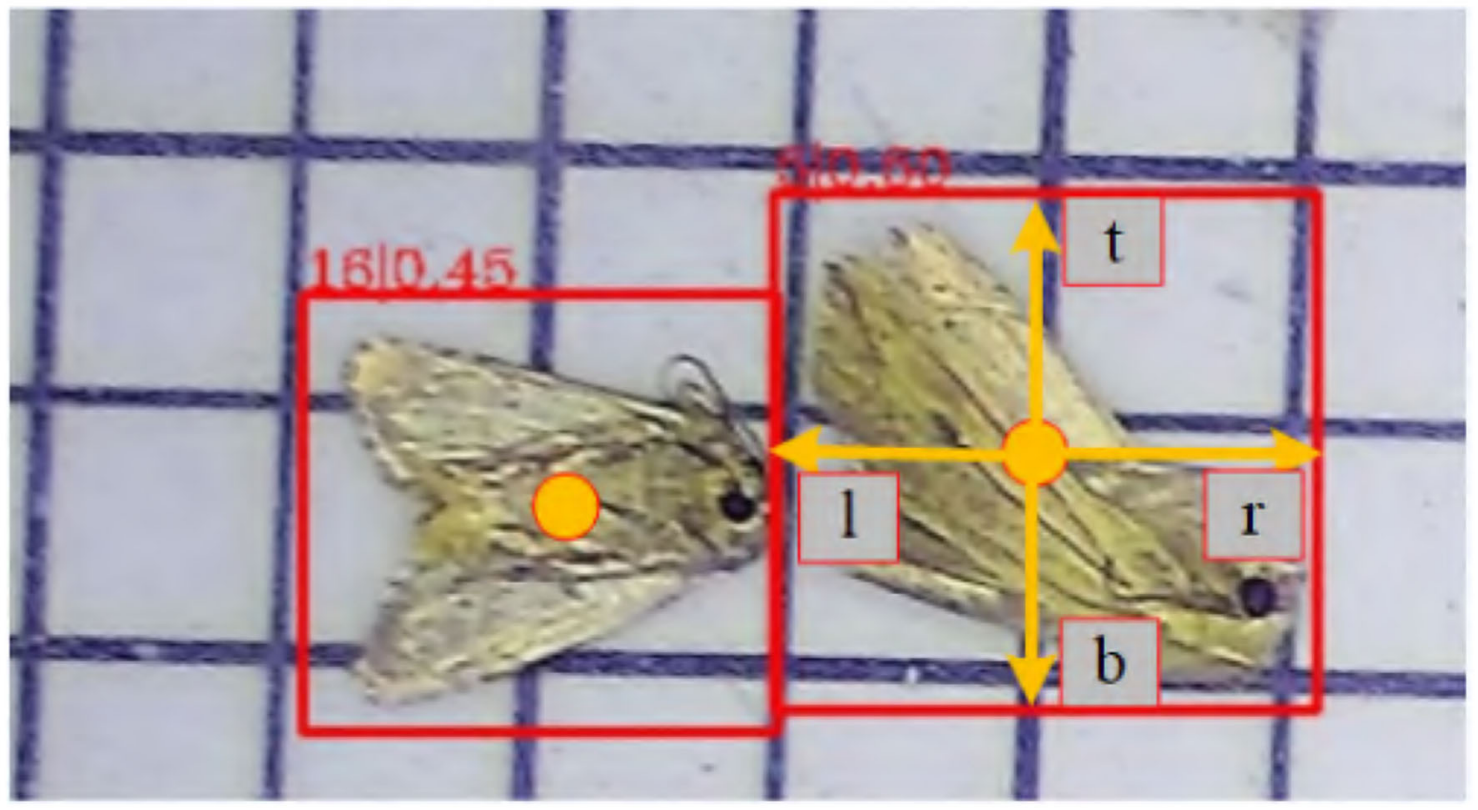
ASP-Det works by predicting a 4D vector (l,t,r,b) encoding the location of a bounding box at each foreground pixel.

In addition, we observed that it is due to many low-quality predicted bounding boxes produced by locations far away from the center of an object. We propose a simple yet effective strategy to suppress these low-quality detected bounding boxes without introducing any hyper-parameters. Specifically, we add a single layer branch in parallel with the regression branch to predict the center-ness of a location, as shown in Figure 8. Given the regression targets l, t, r, and b for a site, the center-ness target is defined as,


(23) $$\begin{array}{l} center-ness=\sqrt{\frac{min\left(l,r\right)}{max\left(l,r\right)}\times \frac{min\left(t,b\right)}{max\left(t,b\right)}} \end{array}$$

We define our training loss function as follows:


(24) $$\begin{array}{l} L\left(p_{x,y},q_{x,y},O_{x,y}\right)=\frac{1}{N_{pos}}\underset{x,y}{\sum }L_{cls}\left(p_{x,y},c_{x,y}^{*}\right)\\ \;+\lambda_{1}\frac{1}{N_{pos}}\underset{x,y}{\sum }\text{sign}\left(c_{x,y}^{*}>0\right)L_{reg}\left(q_{x,y},q_{x,y}^{*}\right)\\ \;+\lambda_{2}L_{centerness}\left(O_{x,y},O_{x,y}^{*}\right) \end{array}$$

where *L*_*cls*_ is the focal loss as in Lin et al. (2017c), *L*_*reg*_ is the IOU loss as in UnitBox (Yu et al., 2016), and *L*_*centerness*_ is the center-ness loss ranges from 0 to 1 and is thus trained with binary cross entropy (BCE) loss. *N*_*pos*_ denotes the number of positive samples and the summation is calculated over all locations on the feature maps *F*_*i*_. The indicator function being 1 if $c_{x,y}^{*}>0$ otherwise is 0. The balanced parameter λ_1_ and λ_2_ are set to 1. We employ sqrt here to slow down the decay of the center-ness. When testing, the final score *S*_*x,y*_ (used for ranking the detections in NMS) is the square root of the product of the predicted center-ness *O*_*x,y*_ and the corresponding classification score *P*_*x,y*_. After the above center-ness suppression, we can obtain better pest detection performance.


(25) $$\begin{array}{l} S_{x,y}=\sqrt{P_{x,y}\times \;O_{x,y}} \end{array}$$

## 6. Experiments

### 6.1. Experiment Settings

#### 6.1.1. Evaluation Metrics

In this paper, we apply five metrics to evaluate the performance of our similar pest detection method: AP50 (Precision in 0.5), AP75 (Precision in 0.75), mAP (mean Average Precision), Recall and MR (mean Recall), and BPR (Best Possible Recall).

#### 6.1.2. Training Details

ResNet-50 is used as our backbone network, and the same hyper-parameters with FCOS are used. Specifically, our network is trained with stochastic gradient descent (SGD) for 90 k iterations with the initial learning rate is 0.0125 and a mini-batch of four images. We trained the network for 12 epochs, ran SGD for the first eight epochs, reduced the learning rate to one-tenth in the 11th epoch, and reduced the learning rate to one-tenth in the 11th epoch. We initialize our backbone networks with the weights pre-trained on ImageNet (Jia et al., 2009). For the newly added layers, we initialize them as in Lin et al. (2017c).

#### 6.1.3. Inference Details

We first forward the input image through the network and obtain the predicted bounding boxes with the predicted class scores. The next post-processing of ASP-Det strictly follows that of FCOS. The post-processing hyper-parameters are also the same, except we use NMS threshold of 0.5 instead of 0.6 in FCOS. Moreover, we use the exact sizes of input images as in training.

### 6.2. Pest Detection Performance of ASP-Det

The section shows that the concern is not particularly important by comparing the MR of ASP-Det and that of its anchor-based counterpart on the dataset. The following analyses are based on the ASP-Det implementation in mmdetection2.

#### 6.2.1. Mean Recall (MR) Performance

Formally, MR is defined as the ratio of the number of ground-truth boxes that a detector can recall at the average to the number of all ground-truth boxes. A ground-truth box is recognized if the box is assigned to at least one training sample (i.e., a location in ASP-Det or other detectors), and a training sampling can be associated with at least one ground-truth box. As shown in Table 6, both with a NFP, a SCC, and Center-ness Loss (CL) on reg obtain similar *MR*(58.8*vs*.62.3%), 12.1 points higher than YOLOv3, 5.3 points higher than Faster R-CNN, and 3.5% higher than FCOS. Moreover, because the best recall of current detectors is much lower than 90%, the small Best Possible Recall gap (<1%) between ASP-Det(NFP), ASP-Det(NFP+SCC), and ASP-Det will not affect the performance of a detector. Therefore, the concern about the low Best Possible Recall may not be necessary for our method.

**Table 6:** The MR and BPR for Ablation study for different strategies of assigning objects to FPN levels.

**Methods**	**PSA**	**NFP**	**CL**	**SCC**	**MR**	**BPR**
Faster R-CNN					57.0	87.2
YOLOv3					50.2	88.9
FCOS					58.8	88.7
ATSS					61.4	93.6
Swin-t					61.8	93.7
ASP-Det (ours)	√				62.2	91.9
ASP-Det (ours)	√	√			62.3	93.5
ASP-Det (ours)	√	√	√		62.4	94.3
ASP-Det (ours)	√	√	√	√	62.3	94.5

#### 6.2.2. Average Precision (AP) Performance

To test the effectiveness of our ASP-Det, we compare the quality pest bounding box by ASP-Det and other state-of-the-art detectors. We choose faster R-CNN, FCOS, and YOLOv3 to compare our proposed ASP-Det on a similar pest dataset. The pest detection results are shown in Tables 7, 8. We can observe that our method outperforms faster R-CNN and YOLOv3. The mAP of our method can achieve 45%, 14.2 higher than YOLOv3, and 3.1 higher than Faster R-CNN. For extreme special pests (classes “21” and “23”), the detection accuracy is lower than other classes of pests. However, our method still performs better than YOLOv3 and Faster R-CNN, benefiting from our feature fusion module.

**Table 7:** Overall performance comparison.

**Method**	**PSA**	**NFP**	**CL**	**SCC**	**AP**	***AP*_**50**_**	***AP*_**75**_**
**General object detection**							
Faster R-CNN (Ren et al., 2015)					41.9	70.7	46.2
YOLOv3 (Redmon and Farhadi, 2018)					30.8	63.2	25.1
FCOS (Tian et al., 2019)					44.0	73.0	49.0
ATSS (Zhang et al., 2020)					44.2	73.0	49.0
Swin-t (Liu Z. et al., 2021)					43.6	74.1	47.2
**Pest sdetection**							
AF-RCNN (Jiao et al., 2020)					31.6	50.3	32.6
PestNet (Zhang et al., 2020)					42.1	70.9	36.3
**Ours**							
ASP-Det	√				44.1	73.2	49.2
ASP-Det	√	√			44.3	73.6	49.4
ASP-Det	√	√	√		44.6	74.3	49.9
ASP-Det	√	√	√	√	**45.0**	**74.9**	**50.2**

*Boldface represents emphasis*.

**Table 8:** AP50 and all classes of pests for different detection methods on the similar pest dataset.

**Pest ID**	**YOLOv3**	**Faster R-CNN**	**FCOS**	**ATSS**	**Swin**	**ASP-Det (ours)**
1	55.6	64.7	71.2	73.2	73.6	**73.9**
2	56.0	65.2	68.5	70.9	70.8	**70.9**
3	67.9	72.0	75.3	75.6	76.4	**76.6**
4	64.1	72.3	69.0	72.5	72.6	**73.3**
5	73.0	79.1	81.4	81.4	81.5	**81.6**
6	85.8	88.3	90.1	**90.2**	89.9	90.0
7	75.7	78.7	81.0	81.4	81.5	**81.6**
8	72.6	76.2	78.7	78.4	**78.8**	**78.8**
9	59.0	77.6	77.7	**82.1**	81.5	81.6
10	65.4	72.6	75.2	76.8	76.9	**77.0**
11	52.6	57.4	60.0	61.6	61.2	**62.3**
12	74.3	79.5	82.1	82.9	**83.4**	82.6
13	75.6	85.6	86.6	**87.5**	87.6	87.2
14	38.1	62.7	67.8	66.5	69.7	**69.8**
15	55.5	66.5	67.9	**69.8**	**69.8**	69.6
16	65.9	74.2	75.7	**76.3**	75.7	75.8
17	54.3	59.4	63.4	**65.4**	64.0	64.1
18	84.2	87.8	89.4	89.3	89.5	**89.6**
19	88.3	90.1	90.3	91.1	91.1	**91.2**
20	94.2	95.5	95.7	95.1	95.1	**95.9**
21	17.5	34.4	46.1	39.7	48.9	**49.0**
22	79.2	82.2	83.4	**85.4**	84.8	85.0
23	27.9	29.4	34.4	31.7	35.4	**36.0**
24	35.6	46.7	50.0	54.1	54.3	**54.4**
mean	63.2	70.7	73.0	74.5	**74.1**	**74.9**

*Boldface represents emphasis*.

**Figure 9:**
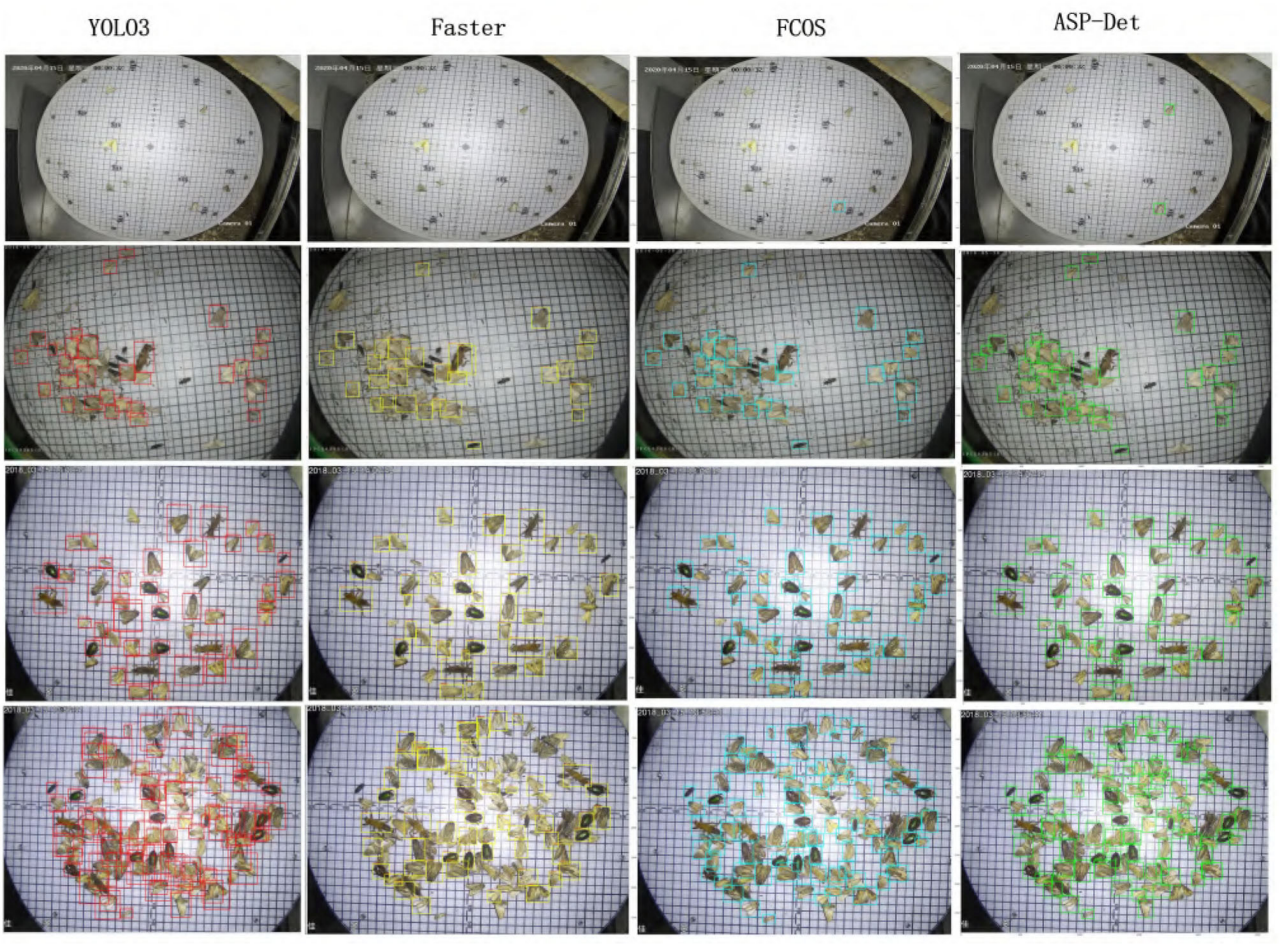
Detection results of YOLOv3 (column 1), Faster RCNN (column 2), FCOS(column 3), and our ASP-Det (column 4).

**Figure 10:**
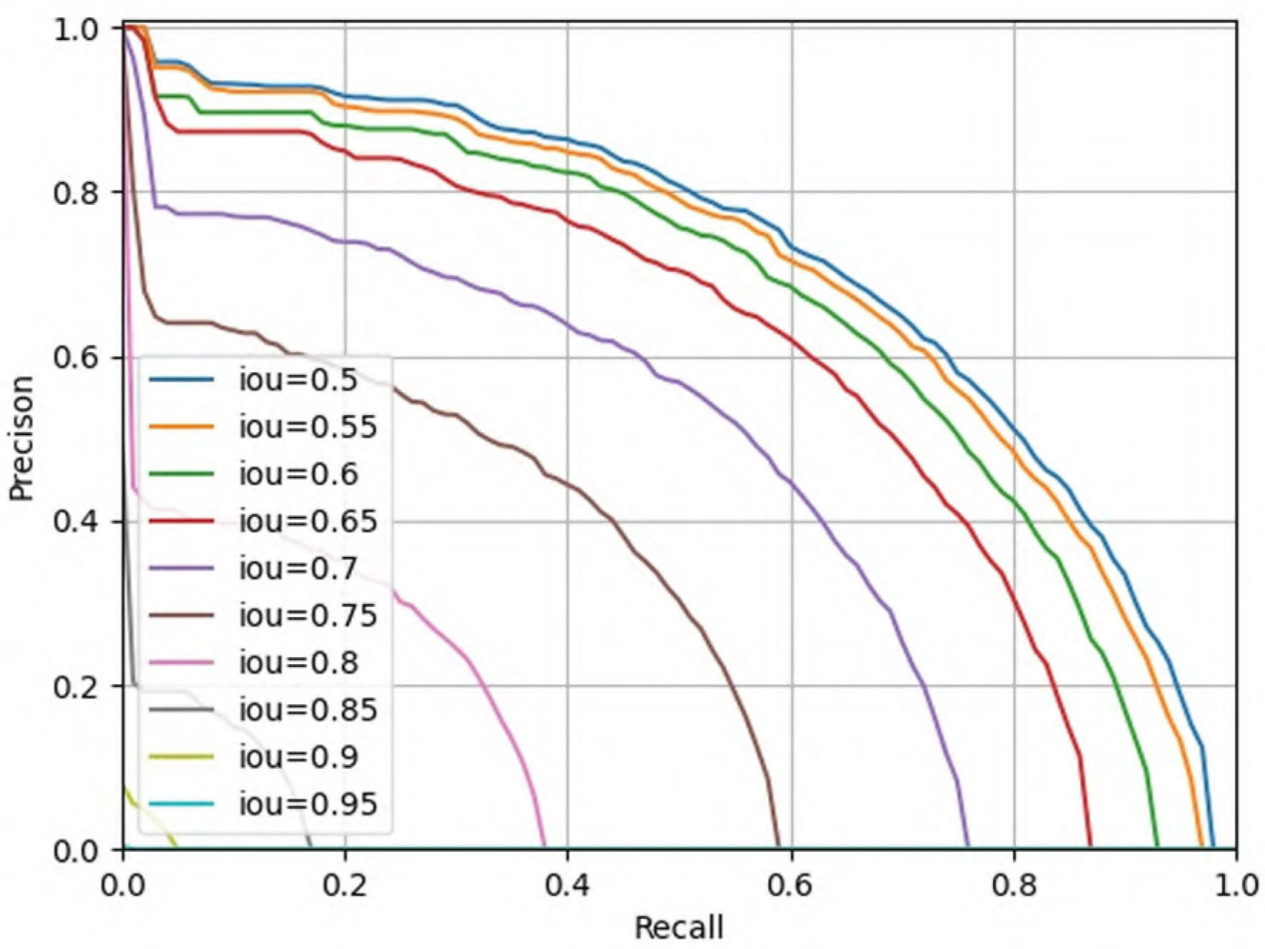
Classification results of IOU (0.5–0.95).

In order to be able to directly observe the advantages of our proposed pest detection method compared with other methods. We show some visualized pest detection results of our practices, YOLOv3 and Faster R-CNN, as shown in Figure 9. It shows that our method can achieve more accurate results and fewer missing pests than the other methods. The model also uses the detection results to graph the classification value and recall rate of IOU in the interval of 0.5 and 0.95 from the Figure 10; our model has good convergence and a high recall rate and accuracy rate.

### 6.3. Ablation Experiments

#### 6.3.1. The Effectiveness of PSA

A PSA mechanism introduces, which prevents background noises, and refines similar pest features. The self-attention module uses novel vector attention, generating content adaptation ability while maintaining the channel adaptation ability. The self-attention module makes our similar target detection model have strong adaptability, effectively removing and enhancing the salient differences between different features. The PSA mechanism is beneficial for feature extraction of objects with appearance-similar. We introduce the PSA mechanism to obtain the weights for each channel and multiply them with the raw feature map.

#### 6.3.2. The Effectiveness of SCC

Because some pests are highly similar in appearance and almost the same size, in the training process, we deal with the ambiguity of the same FPN level by selecting the bounding box with the smallest area. In the test, if two objects A and B with the same category overlap, no matter which objects the position in the overlap prediction is, the forecast is correct. The missing object can be predicted by the work only belonging to it. If A and B do not belong to the same category, the overlapping position may indicate the category of A but will return to the bounding box of B, which will cause errors. The SCC module is mainly used to adjust the size jump problem in FPN. Using the SCC module can make the pests have a larger field of vision in feature areas of similar sizes, which helps distinguish the illusion of classification confusion caused by similar texture problems.

#### 6.3.3. The Effectiveness of Center-Ness

ASP-Det using multi-level FPN prediction can only solve the target occlusion between different sizes. In the same feature-level processing, intractable ambiguity will still appear. However, the size of most of the target data in our dataset is not much different. Many of these problems that need to be considered are the occlusion problems of targets of the same scale. As mentioned before, we introduce center-ness to suppress the low-quality detected bounding boxes produced by the locations far from the center of an object. As shown in Table 7, the center-ness branch is used in regression and classification. The AP improvement of the dataset is not very large; AP from 44.3 to 44.6% is not obvious.

#### 6.3.4. The Effectiveness of Different Backbones

To prove that our module plays a vital role in different backbones, we use several backbone frameworks for experiments, as shown in the Table 9. Our proposed method has good performance for our proposed ASPD task, so applications that expect the same task can refer to and use this algorithm framework. Using different backbones for ASPD tasks, from the results, the resnet network structure is more mature and robust, and the accuracy is higher. Without a better and faster implementation method, it is relatively safe to use the resnet network architecture at the current practical stage.

**Table 9:** The ap value for Pest-as under different backbones.

**Backbone**	***AP***	***AP*_**50**_**	***AP*_**75**_**
ResNet-50	45.0	74.9	50.2
HRnet	44.6	74.4	49.9
ResNetXt	45.5	75.5	50.9
Res2Net	45.1	74.6	50.2
Swin-t transform	44.6	74.9	48.2

### 6.4. Real-Time Performance

**Figure 11:**
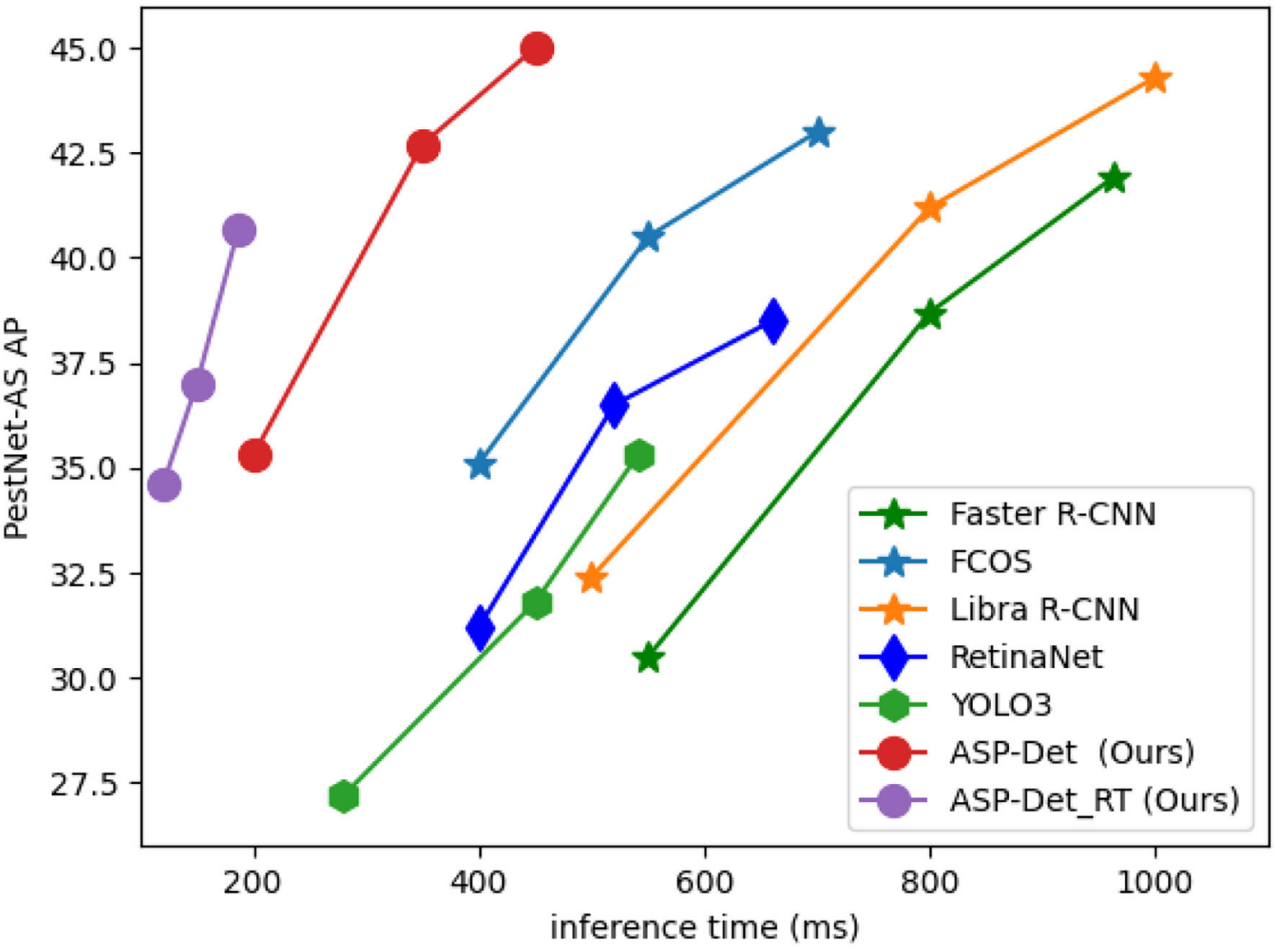
Comparisons of efficient of different modules proposed in this paper with the-state-of-arts method on similar pest dataset on a single GPU.

In the field of real-time image enhancement, image super-resolution (SR) is a crucial research hotspot (Liu X. et al., 2021). In real-time applications in agriculture, real-time performance is also critical. Real-time depth models are prominent in practical applications as an agricultural image detection method. Moreover, we also designed a real-time version named ASP-Det_RT. We reduce the scale of input images from 1,333 × 800 to 800 × 512, which decreases the inference time per image by 50%. The effect is shown in Figure 11.

We evaluate the computation efficiency of our multi-categories similar pest detector from the aspects of training and testing time and compare it with FCOS, YOLOv3, and Faster R-CNN. The testing time of our method and FCOS method takes 0.045 s per pest image in total, which is slightly faster than Faster R-CNN and 2.5 times slower than the YOLOv3 detector. However, compared with FCOS and YOLOv3 detectors, the training time of our pest detector is faster, and most importantly, the detection precision of our approach is primarily higher than YOLOv3. Otherwise, the hyper-parameter of our approach is less than Faster R-CNN and YOLOv3. Therefore, considering detection efficiency and accuracy, our method is the best choice and applicable to detect the 24-category similar pests.

### 6.5. Qualitative Results

**Figure 12:**
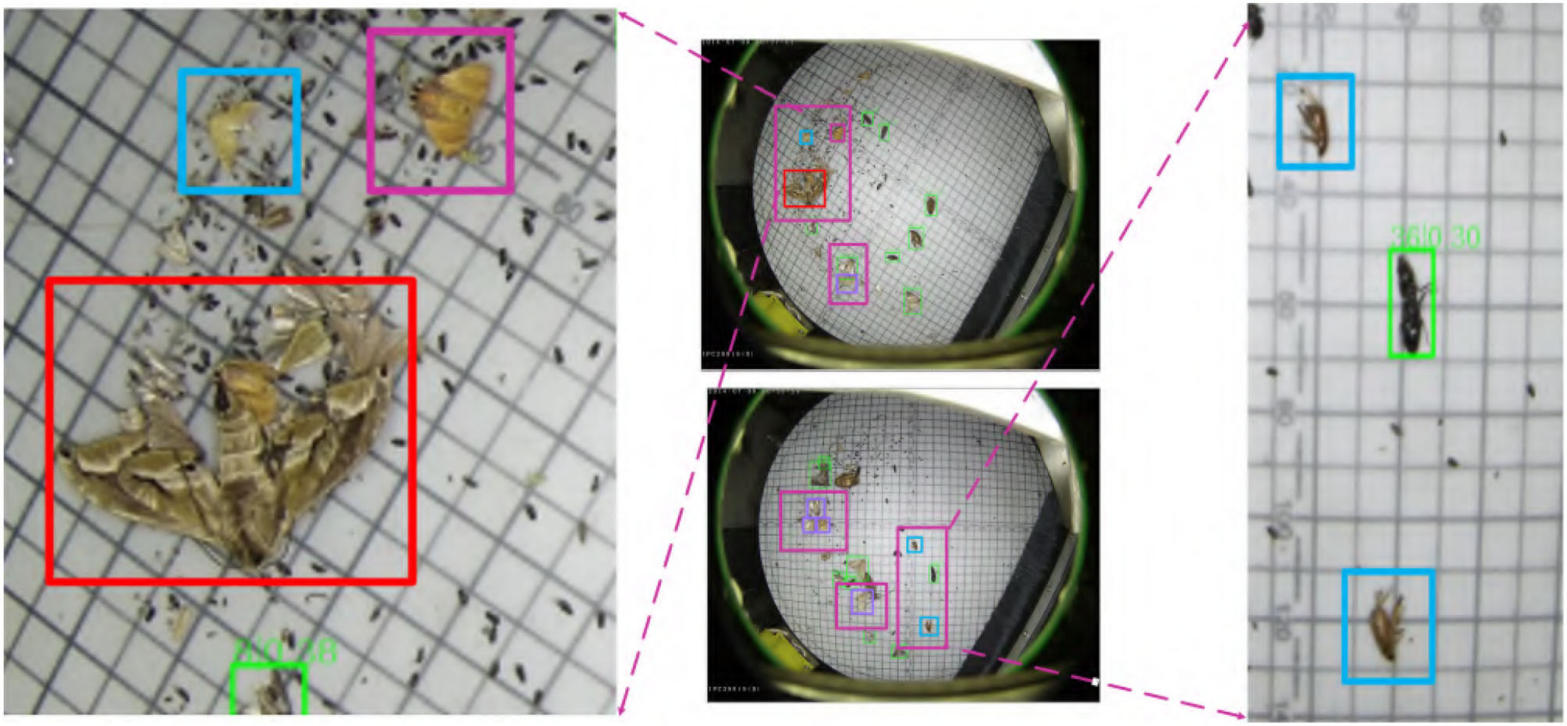
Some problems in the ASPD-Det detection method, misclassification, or omission of detection.

For appearance-similar agricultural pests, even if we use the attention mechanism, non-local fusion, and skip module for processing, the target still has some misclassifications and undetectable situations. As shown in Figure 12, other pests located around the larger size pests inside the red box are difficult to identify and may be affected by the size and posture of the pests in the box. Another part is due to the problem of the time interval for catching pests, which causes some distortion of the color of some pests (the pink boxes) and misses inspection. The model may not recognize some pests because they are too similar to the background color or neighboring pests (like the sample in the purple box in the first image). Another part is that the size of the pests is relatively small compared to the original size in other pictures, and the posture is also more diverse, which causes the model to miss detection (such as the sample in the cyan box). Finally, there may be missed detection due to the model's limitations, which will be the main focus of follow-up research.

## 7. Conclusion

Our proposed ASP-Det does not employ IoU scores between anchor and ground-truth boxes to determine the training labels. Additionally, ASP-Det avoids all computation and hyper-parameters related to anchor boxes and solves similar pest detection in a per-pixel prediction fashion, similar to other dense prediction tasks, such as semantic segmentation. Fortunately, the accuracy of ASP-Det is also excellent for pest appearance-similarity. Given the superior performance and merits of the anchor-free detector (e.g., much more straightforward and fewer hyper-parameters), we encourage plant protection to rethink the necessity of anchor boxes in object detection. Additionally, to apply our pest detection method in practice, we present some real-time models of our detector, which has excellent performance and inference speed. Given its effectiveness and efficiency, we hope that ASP-Det can serve as a solid and straightforward alternative for promoting agricultural production.

## Data Availability Statement

The data analyzed in this study is subject to the following licenses/restrictions: The data set was provided by Jiaduo Company, which is a cooperative unit of our research institution. The disclosure of the data requires the consent of the company before it can be released to the public. Requests to access these datasets should be directed to wangfenmei205@126.com.

## Author Contributions

FW is responsible for overall model building, paper writing, and dataset training and modeling. LL is responsible for guiding the writing of the thesis and the construction of the overall architecture. SD is responsible for model optimization and code debugging. SW is responsible for the derivation and verification of the model formula. ZH only needs to participate in the drawing work. HH is responsible for the curation and fabrication of the dataset. JD gave a lot of guidance in the revision process of the paper, and proofread the full text. All authors contributed to the article and approved the submitted version.

## Funding

This work was supported in part by the project of the Dean's Fund of Hefei Institute of Physical Science, Chinese Academy of Sciences (YZJJ2022QN32) and the major special project of Anhui Province Science and Technology (2020b06050001).

## Conflict of Interest

The authors declare that the research was conducted in the absence of any commercial or financial relationships that could be construed as a potential conflict of interest.

## Publisher's Note

All claims expressed in this article are solely those of the authors and do not necessarily represent those of their affiliated organizations, or those of the publisher, the editors and the reviewers. Any product that may be evaluated in this article, or claim that may be made by its manufacturer, is not guaranteed or endorsed by the publisher.

## Footnotes

^1^The PestNet is a set of light trap datasets jointly annotated by professionals and agricultural experts from Jiaduo Company, which provides data support for intelligence agriculture. Artificial Intelligence Agriculture Valley has developed a special labeling software for agricultural pests and diseases. This dataset is also selected and organized in this dataset driven by similar pest detection problems.
